# All for One and One for All? – Examining Convergent Validity and Responsiveness of the German Versions of the Tinnitus Questionnaire (TQ), Tinnitus Handicap Inventory (THI), and Tinnitus Functional Index (TFI)

**DOI:** 10.3389/fpsyg.2021.596037

**Published:** 2021-03-12

**Authors:** Benjamin Boecking, Petra Brueggemann, Tobias Kleinjung, Birgit Mazurek

**Affiliations:** ^1^Charité – Universitätsmedizin Berlin, Berlin, Germany; ^2^Department of Otorhinolaryngology, University Hospital of Zürich, Zurich, Switzerland

**Keywords:** tinnitus, tinnitus questionnaire, tinnitus functional index, tinnitus handicap inventory, treatment responsiveness, convergent validity, responsiveness

## Abstract

**Background:**

Measurement of tinnitus-related distress and treatment responsiveness is key in understanding, conceptualizing and addressing this often-disabling symptom. Whilst several self-report measures exist, the heterogeneity of patient populations, available translations, and treatment contexts requires ongoing psychometric replication and validation efforts.

**Objective:**

To investigate the convergent validity and responsiveness of the German versions of the Tinnitus Questionnaire [TQ], Tinnitus Handicap Inventory [THI], and Tinnitus Functional Index [TFI] in a large German-speaking sample of patients with chronic tinnitus who completed a psychologically anchored 7-day Intensive Multimodal Treatment Programme.

**Methods:**

Two-hundred-and-ten patients with chronic tinnitus completed all three questionnaires at baseline and post-treatment. Intraclass correlation coefficients determined the convergent validity of each questionnaire’s total and subscale scores. Treatment responsiveness was investigated by [a] comparing treatment-related change in responders vs. non-responders as classified by each questionnaire’s minimal clinically important difference-threshold, and [b] comparing agreement between the questionnaires’ responder classifications.

**Results:**

The total scores of all three questionnaires showed high agreement before and after therapy (TQ | THI: 0.80 [Pre], 0.83 [Post], TQ | TFI: 0.72 [Pre], 0.78 [Post], THI | TFI: 0.76 [Pre] 0.80 [Post]). All total scores changed significantly with treatment yielding small effect sizes. The TQ and TFI yielded comparable (19.65 and 18.64%) and the THI higher responder rates (38.15%). The TQ | THI and TQ | TFI showed fair, and the THI | TFI moderate agreement of responder classifications. Independent of classification, responders showed significantly higher change rates than non-responders across most scores. Each questionnaire’s total change score distinguished between responders and non-responders as classified by the remaining two questionnaires.

**Conclusion:**

The total scores of all three questionnaires show high convergent validity and thus, comparability across clinical and research contexts. By contrast, subscale scores show high inconsistency. Whilst the TFI appears well suited for research purposes, the THI may be better suited to measure psychological aspects of tinnitus-related distress and their changes with accordingly focused treatment approaches.

## Introduction

Subjective tinnitus is a multicausally generated symptom that denotes an auditory “phantom” perception without external sound source. Prevalence estimates vary widely due to broad variations in study quality and construct definitions and range between 5 and 43% (McCormack et al., 2016; Biswas and Hall, 2020). The majority of people habituate to the percept (Phillips et al., 2018). However, a subset of those affected link its experience to the onset or exacerbation of psychological distress (Langguth et al., 2013) which may pose a key risk factor for symptom chronification (Wallhäusser-Franke et al., 2017) and can severely impact upon individuals’ quality of life (Cima et al., 2011; Baguley et al., 2013; Ayodele et al., 2021).

In conceptualizing the interplay between tinnitus and affective symptomatology, many researchers have highlighted interdependent associations between tinnitus-related- and broader psychological distress (Ahmed et al., 2017; Bhatt et al., 2017; Boecking et al., 2019). Hence, it is not surprising that psychological treatment approaches have demonstrated effectiveness across both tinnitus-specific and associated psychological domains (Hesser et al., 2011; Cima et al., 2014; Zenner et al., 2017; Landry et al., 2020). Among these treatment approaches, a 7-day Intensive Multimodal Therapy Programme has demonstrated beneficial, if small, long-term effects on tinnitus-related distress, wider emotional distress, and depressive symptoms (Seydel et al., 2010, 2015; Brueggemann et al., 2018a,b). This psychologically anchored, 7-day Intensive Multimodal Therapy Programme comprised detailed ear-nose-throat (ENT), psychosomatic and psychological diagnostics as well as psychoeducational (“counseling”), auditory, relaxation and physiotherapy-related elements whilst placing particular emphasis on cognitive-behavioral treatment components to address and alleviate emotional distress.

Given that there are no objective measures of tinnitus to date (Jackson et al., 2019), researchers and clinicians can choose from different self-report questionnaires to evaluate patients’ tinnitus-related distress and its potential amelioration with different treatment approaches. Commonly used measures comprise the Tinnitus Questionnaire (TQ; Hallam, 1996; German version: Goebel and Hiller, 1998), Tinnitus Handicap Inventory (THI: Newman et al., 1996, 1998; German version: Kleinjung et al., 2007), or Tinnitus Functional Index [TFI: (Meikle et al., 2012; Henry et al., 2016; German version: Brueggemann et al., 2017)].

These measures differentially weigh tinnitus-specific, psychological, auditory or health-related aspects of tinnitus-related distress (Hall et al., 2016) and numerous translations have been validated [e.g., Dutch (Meeus et al., 2007; Rabau et al., 2014), Swedish (Müller et al., 2016), Persian (Mahmoudian et al., 2011), Danish (Zachariae et al., 2000), Chinese (Wang et al., 2020), or Polish (Wrzosek et al., 2016) amongst others]. Within this measurement landscape, there have been calls for a harmonization of measures to facilitate standardization and comparability of both construct operationalizations and treatment effects (Hall et al., 2016). For example, previous work demonstrated acceptable-to-high convergent validity between the total scores of the TQ | THI (Baguley et al., 2000; Robinson et al., 2003; Zeman et al., 2012), TFI | THI (Fackrell et al., 2016), and TQ | TFI, respectively (Jacquemin et al., 2019). Investigating the questionnaires’ performances across different timepoints, only minor differences were reported when examining [a] test-retest statistics for the TQ and THI (Baguley et al., 2000; Zeman et al., 2012) or [b] the effects of a High-Definition transcranial Direct Current Stimulation (HD-tDCS) treatment on the TQ and TFI (HD-tDCS; Jacquemin et al., 2019).

Amidst these psychometric evaluation efforts, replications of previous findings are essential in order to build a reliable and valid evidence base across measures, translations, patient populations and treatment approaches (Zeman et al., 2012; Fackrell et al., 2016). For example, it has been pointed out that the English and German versions of the TQ differ considerably (Fackrell et al., 2014) thus warranting investigations of its convergent validity and responsiveness in a German population.

The present study adds to this literature by being the first to investigate, in the same study, the convergent validity and treatment responsiveness of the German versions of the Tinnitus Questionnaire [TQ], Tinnitus Handicap Inventory [THI], and Tinnitus Functional Index [TFI] in a large convenience sample of patients with chronic tinnitus who completed a psychologically anchored, 7-day Intensive Multimodal Therapy Programme.

## Materials and Methods

### Participants

The present sample consisted of *N* = 210 adult patients with chronic tinnitus who attended the Tinnituscentre in 2015 and provided both baseline and post-treatment data; i.e., completed the TQ, THI, and TFI on the first and last days of the therapy programme. Participants were between 18 and 77 years old (*M*_*age*_ = 48.39 years; *SD* = 12.38). Forty-four percent were female.

Participants were included if they were 18 years of age or older and reported experiencing chronic tinnitus for more than 3 months. Subjects were excluded if they reported significant difficulties in understanding the German language or if identifiable medical factors explained the tinnitus symptomatology. All patients signed an informed consent form agreeing for the study data to be collected and used for research purposes. The Charité Universitätsmedizin Berlin’s ethics committee approved data analysis (EA4/137/20).

### Measures

#### Tinnitus Questionnaire (German version)

The TQ (Hallam, 1996; German version: Goebel and Hiller, 1998) is a self-report measure designed to assess *tinnitus-related distress*. The German version consists of 52 statements that are answered on a 3-point scale (0 = not true, 1 = partly true; 2 = true). The total score sums 40 items with two items being included twice, thus yielding a score between 0 and 84. The TQ comprises six sub-scales: [1] cognitive and [2] emotional distress, [3] intrusiveness, [4] auditory perceptual difficulties, [5] sleep disturbances, and [6] somatic complaints. The subscales are not validated for diagnostic assessment or the measurement of treatment-related change. A minimal clinically important difference (MCID) of 12 points has been considered to denote reliable clinically significant improvement (Hall et al., 2018). In the current sample, the measure’s internal consistency was excellent (α = 0.92).

#### Tinnitus Handicap Inventory

The THI (Newman et al., 1996, 1998; German version: Kleinjung et al., 2007) measures self-perceived *tinnitus handicap severity*. It consists of 25 items that are answered on a 3-point scale (0 = no; 2 = sometimes; 4 = yes) resulting in a total score between 0 and 100. It features three subscales: [1] functional (role limitations in the areas of mental, social/occupational, and physical functioning), [2] emotional (affective reactions to tinnitus), and [3] catastrophic responses [catastrophic responses to the symptoms of tinnitus; (Newman et al., 1996, p. 144)]. A change score of at least seven points has been considered to denote reliable clinically significant improvement (Zeman et al., 2011). In the current sample, the measure’s internal consistency was excellent (α = 0.93).

#### Tinnitus Functional Index

The TFI (Meikle et al., 2012; Henry et al., 2016; German version: Brueggemann et al., 2017) measures *negative tinnitus impact*. It consists of 25 items that are answered on a 10-point Likert scale. Sum scores are linearly transformed to range from 0 to 100. The items load on eight subscales: [1] intrusive (unpleasantness, intrusiveness, persistence), [2] sense of control (reduced sense of control), [3] cognitive (cognitive interference), [4] sleep (sleep disturbance), [5] auditory (auditory difficulties attributed to tinnitus), [6] relaxation (interference with relaxation), [7] quality of life (quality of life reduced), and [8] emotional (emotional distress). A change score of at least 13 points has been considered to denote reliable clinically significant improvement (Meikle et al., 2012). In the current sample, the measure’s internal consistency was excellent (α = 0.97).

### Statistical Analysis

Where possible, statistical analyses for this paper follow the approach applied and reported by Jacquemin et al. (2019) in order to facilitate comparability of results. Importantly, however, whilst Jacquemin et al. (2019) examine the TQ and TFI’s responsiveness to HD-tDCS-treatment with regard to an additionally measured external criterion (a patient-rated clinical global improvement score), such a criterion was not available in the present study. Hence, the here-reported responsiveness analyses are limited to three-way cross-comparisons.

#### Descriptive Analyses and Treatment-Related Changes

We examined the means and standard deviations for the tinnitus and psychological measures at baseline and post-treatment. Treatment-related change was quantified by computing dependent samples *t*-tests and estimating effect sizes *d* with 95% confidence intervals (Cohen, 1988). Here, | *d* | < 0.20 denotes a negligible, 0.20 < | *d* | < 0.49 a small, 0.50 < | *d* | < 0.79 a moderate, and | *d* | > 0.80 a large effect size.

#### Convergent Validity

Convergent and discriminant validity between the tinnitus questionnaires’ total and subscale scores was examined using two-way-mixed intra-class correlation coefficients (*ICC*; Field, 2005). We expected high convergent validity between the tinnitus questionnaires’ total scores. By contrast, expectations for the subscale scores were varied (Jacquemin et al., 2019). *ICC* coefficients of <0.50 indicate poor, 0.50 < *ICC* < 0.75 moderate, 0.76 < *ICC* < 0.90 good, and *ICC* > 0.91 excellent agreement (Koo and Li, 2016).

#### Responsiveness

Based on the respective tinnitus questionnaires’ minimal clinically significant improvement thresholds, patients were classified as responders or non-responders. To compare the questionnaires’ responder classifications, we computed three sets of analyses: First, κ coefficients indexed the agreement between the different responder classifications (Altman, 1991). κ < 0.00 indicates poor, 0.00 < κ < 0.20 slight, 0.21 < κ < 0.40 fair, 0.41 < κ < 0.60 moderate, 0.61 < κ < 0.80 substantial, and κ > 0.81 perfect agreement (Landis and Koch, 1977). Second, independent samples *t*-tests compared change scores between the respectively classified responder vs. non-responder subgroups across both total and subscale scores of each tinnitus questionnaire. Third, Receiver Operator Characteristics (*ROC*) analyses investigated, if each tinnitus questionnaire’s [a] change or [b] post-treatment score effectively distinguished between responders and non-responders as classified by the two respectively remaining questionnaires. The associated “area under the curve” statistic (*AUC*) denotes 0.50 < *AUC* < 0.70 low, 0.71 < *AUC* < 0.90 moderate, and *AUC* > 0.91 high ability of the predictor variable to do so (Streiner and Cairney, 2007; Pintea and Moldovan, 2009). All analyses were computed using SPSS statistical software version 25 (SPSS Inc., Chicago Il, United States).

## Results

### Descriptive Analyses and Treatment-Related Changes

**FIGURE 1:**
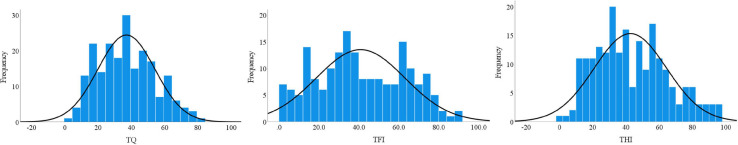
Frequency distributions of the total scores of the tinnitus measures at baseline: TQ (*M* = 37.10, *SD* = 17.15, *n* = 210), THI (*M* = 42.70, *SD* = 21.79, *n* = 209), and TFI (*M* = 40.89, *SD* = 22.20, *n* = 188). TQ, tinnitus questionnaire [German version]; THI, tinnitus handicap inventory; TFI, tinnitus functional index.

As a first step, the frequency distributions of the total scores of the three tinnitus questionnaires were examined at baseline (see Figure 1). Visual inspection of the associated Q–Q plots suggested that the tinnitus scores were normally distributed, and we used parametric tests for all subsequent analyses.

Investigating treatment-related change, Table 1 features descriptive statistics and baseline-to-post-treatment changes for each questionnaire’s total and subscale scores.

**TABLE 1:** Means, standard deviations, and treatment-related changes for the tinnitus questionnaires.

	**Baseline**		**Post treatment**		**Dependent samples *t*-test**		**Cohen’s *d*: 95% CI**	
	***Mean***	***SD***	***Mean***	***SD***	***t(df)***	***p***	***LLCI***	***ULCI***
**TQ**								
Total	37.10	17.15	30.68	16.75	8.43 (172)	0.00	0.29	0.46
Emotional distress	10.20	5.51	8.00	5.45	8.88 (172)	0.00	0.29	0.47
Cognitive distress	6.55	4.11	4.72	3.85	8.21 (172)	0.00	0.29	0.49
Intrusiveness	9.70	3.84	8.37	3.93	7.42 (172)	0.00	0.24	0.43
Auditory perceptual difficulties	5.00	3.77	4.39	3.73	2.63 (172)	0.09	0.03	0.20
Sleep disturbances	3.50	2.51	3.01	2.47	3.55 (172)	0.01	0.06	0.23
Somatic complaints	2.14	1.83	2.19	1.89	−1.73 (172)	n.s.	−0.19	0.01
**THI**								
Total	42.70	21.79	35.80	22.36	6.47 (170)	0.00	0.20	0.39
Catastrophic	4.47	2.31	3.24	2.44	8.05 (170)	0.00	0.36	0.62
Emotional	6.67	4.72	5.69	4.78	4.36 (170)	0.00	0.11	0.31
Functional	10.21	5.04	8.97	5.03	4.98 (170)	0.00	0.13	0.31
**TFI**								
Total	40.89	22.20	34.60	20.39	3.01 (130)	0.00	0.07	0.35
Intrusiveness	54.38	26.44	51.84	23.39	0.52 (130)	n.s.	−0.12	0.21
Control	51.38	28.19	44.48	26.11	2.23 (130)	0.27	0.02	0.38
Cognitive	35.98	24.60	32.05	23.69	1.45 (130)	n.s.	−0.04	0.25
Sleep	41.26	33.39	31.95	25.92	2.57 (130)	0.01	0.04	0.35
Auditory	34.06	29.79	30.18	26.59	0.41 (130)	n.s.	−0.11	0.16
Relaxation	49.54	28.39	39.36	25.92	4.10 (130)	0.00	0.17	0.49
Quality of life	31.54	25.38	25.12	24.05	3.19 (130)	0.02	0.07	0.31
Emotional	32.13	24.51	24.94	24.79	2.62 (130)	0.01	0.05	0.34

*TQ, tinnitus questionnaire [German version]; THI, tinnitus handicap inventory; TFI, tinnitus functional index; *SD*, standard deviation; *LLCI*, lower level of confidence interval; *ULCI*, upper level of confidence interval; n.s., not significant; | *d* | < 0.20 = negligible effect; 0.20 < | *d* | < 0.49 = small effect; 0.50 < | *d* | < 0.79 = moderate effect.*

All total scores showed significant improvements with treatment. Similarly, most subscale scores changed significantly except for [TQ] “somatic complaints,” and [TFI] “intrusiveness,” “cognitive interference,” and “auditory difficulties attributed to tinnitus.” Most changes yielded small effect sizes with confidence intervals ranging from *negligible* ([TQ] auditory perceptual difficulties, sleep disturbances; [TFI] emotional, functional; [THI] total, control, sleep, relaxation, quality of life, emotional) to *moderate* ([THI] “catastrophic responses to the symptoms of tinnitus”).

### Convergent Validity

*Intraclass correlation coefficients* examined the convergent and discriminant validity between the tinnitus questionnaires’ [a] total scores at baseline and post-treatment (Table 2, Panel 1) and [b] subscale scores at baseline, respectively (Panel 2).

**TABLE 2:** Convergent validity of the TQ, THI, and TFI total scores at baseline and post-treatment **(A)** and subscale scores at baseline **(B)**.

**(A)**											
**Baseline**						**Post-treatment**						
***ICC* (95% CI)**						***ICC* (95% CI)**						
**TQ-THI**		**TQ-TFI**		**THI-TFI**		**TQ-THI**		**TQ-TFI**		**THI-TFI**	

0.80 (0.67–0.88)		0.72 (0.64–0.79)		0.76 (0.69–0.81)		0.83 (0.70–0.90)		0.78 (0.67–0.85)		0.80 (0.73–0.85)	

**(B)**											

	***ICC* (95% CI)**										
	**THI**					**TFI**					
**TQ**	**Catastrophic**	**Emotional**	**Functional**	**Intrusiveness**	**Control**	**Cognitive**	**Sleep**	**Auditory**	**Relaxation**	**Quality of life**	**Emotional**

Emotional distress	**0.27 (**−**0.09–0.55)**	**0.63 (0.06–0.83)**	**0.72 (0.64–0.78)**	0.05 (−0.05–0.10)	0.06 (−0.05–0.19)	0.13 (−0.06–0.31)	0.07 (−0.04–0.20)	0.08 (−0.04–0.20)	0.07 (−0.05–0.20)	0.16 (−0.04–0.35)	0.16 (−0.04–0.35)
Cognitive distress	**0.51 (0.19–0.70)**	**0.70 (0.62–0.76)**	**0.41 (0.06–0.63)**	0.03 (−0.03–0.11)	0.04 (−0.04–0.14)	0.03 (−0.03–0.11)	0.05 (−0.05–0.15)	0.04 (−0.05–0.14)	0.04 (−0.04–0.13)	0.09 (−0.05–0.24)	0.09 (−0.05–0.24)
Intrusiveness	**0.24 (**−**0.09–0.54)**	**0.46 (0.11–0.66)**	**0.71 (0.64–0.77)**	0.05 (−0.05–0.17)	0.05 (−0.05–0.17)	0.10 (−0.05–0.25)	0.06 (−0.05–0.17)	0.07 (−0.04–0.19)	0.06 (−0.05–0.18)	0.11 (−0.04–0.27)	0.10 (−0.04–0.24)
Auditory perceptual difficulties	0.33 (0.21–0.45)	**0.39 (0.25–0.51)**	**0.40 (**−**0.10–0.70)**	0.28 (−0.02–0.10)	0.03 (−0.03–0.10)	0.07 (−0.05–0.20)	0.03 (−0.05–0.11)	0.10 (−0.05–0.24)	0.03 (−0.04–0.10)	0.09 (−0.05–0.23)	0.06 (−0.05–0.17)
Sleep disturbances	0.34 (0.20–0.46)	0.25 (0.03–0.43)	0.18 (−0.09–0.45)	0.12 (−0.02–0.06)	0.02 (−0.03–0.07)	0.03 (−0.04–0.10)	0.05 (−0.04–0.15)	0.01 (−0.05–0.09)	0.02 (−0.03–0.08)	0.03 (−0.04–0.12)	0.04 (−0.04–0.13)
Somatic complaints	0.22 (−0.04–0.44)	0.19 (−0.06–0.41)	0.11 (−0.07–0.32)	0.01 (−0.02–0.05)	0.01 (−0.03–0.05)	0.02 (−0.04–0.09)	0.01 (−0.05–0.08)	0.02 (−0.05–0.10)	0.01 (−0.03–0.06)	0.03 (−0.04–0.11)	0.04 (−0.04–0.10)
**THI**											
Catastrophic				0.02 (−0.02–0.08)	0.03 (−0.03–0.09)	0.04 (−0.04–0.13)	0.03 (−0.05–0.11)	0.02 (−0.05–0.10)	0.03 (−0.03–0.10)	0.05 (−0.04–0.15)	0.06 (−0.05–0.17)
Emotional				0.03 (−0.04–0.12)	0.04 (−0.04–0.14)	0.09 (−0.06–0.25)	0.06 (−0.05–0.16)	0.05 (−0.05–0.15)	0.05 (−0.05–0.17)	0.13 (−0.06–0.31)	0.13 (−0.05–0.31)
Functional				0.06 (−0.05–0.18)	0.06 (−0.05–0.20)	0.14 (−0.06–0.33)	0.09 (−0.04–0.22)	0.11 (−0.04–0.25)	0.07 (−0.06–0.21)	0.17 (−0.04–0.36)	0.14 (−0.04–0.32)

*TQ, tinnitus questionnaire (German version); THI (tinnitus handicap inventory); TFI, tinnitus functional index. *ICC*, intraclass correlation (0.50–0.75, moderate, 0.76–0.90, good agreement); CI, confidence interval. Bold ICC coefficients indicate moderate agreement (upper level confidence interval >0.50); all other indices show “poor” agreement.*

The total scores of all tinnitus questionnaires showed moderate-to-good agreement at both baseline and post-treatment. The cognitive-emotional subscale scores of the TQ and THI showed moderate agreement. The TFI subscale scores showed poor agreement with both the TQ and THI subscale indices.

### Responsiveness

**FIGURE 2:**
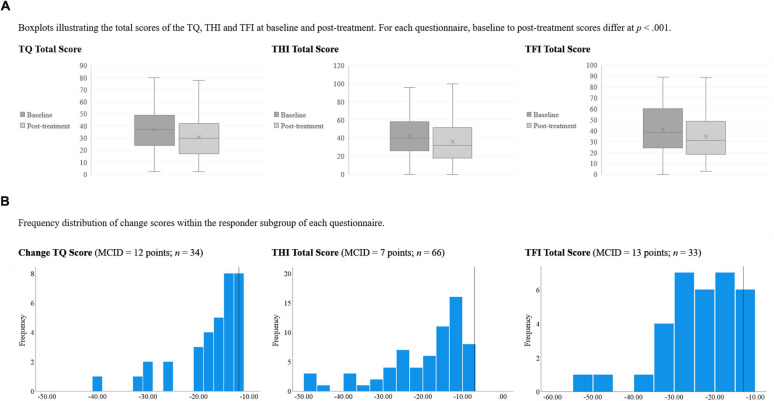
**(A)** Boxplots illustrating the total scores of the TQ, THI, and TFI at baseline and post-treatment. For each questionnaire, baseline to post-treatment scores differ at *p* < 0.001. **(B)** Frequency distribution of change scores within the responder subgroup of each questionnaire. TQ, tinnitus questionnaire [German version]; THI, tinnitus handicap inventory; TFI, tinnitus functional index; MCID, minimal clinically important difference.

Juxtaposing total treatment-related change with that observed in the respectively specified responders subgroups, Figure 2, (A) depicts box plots that illustrate the total scores of the TQ, THI and TFI at baseline and post-treatment (see also Table 1). (B) depicts histograms of the change scores in the respectively classified responder subgroups (as defined by baseline-to-post-treatment change of at least [TQ] 12, [THI] 7, or [TFI] 13 points).

### Comparisons of Change Scores for Responders vs. Non-Responders According to Each Tinnitus Questionnaire’s Responder Classification

Next, we compared the baseline-to-post-treatment change scores for responders vs. non-responders as classified by each questionnaire’s MCID thresholds (Table 3).

**TABLE 3:** Differences in baseline-to-post-treatment change scores for responders vs. non-responders as classified by each tinnitus questionnaire.

	**Responders vs. non-responders classification**								
	**TQ [MCID: −12]**			**THI [MCID: −7]**			**TFI [MCID: −13]**		
**Change scores**	**Responder**	**Non-responder**		**Responder**	**Non-responder**		**Responder**	**Non-responder**	
	**(*n* = 34)**	**(*n* = 139)**	***p***	**(*n* = 66)**	**(*n* = 107)**	***p***	**(*n* = 33)**	**(*n* = 140)**	***p***

**TQ**									
Total	−18.21	−2.47	0.00	−9.18	−3.33	0.00	−11.30	−4.21	0.00
Emotional distress	−5.65	−1.23	0.00	−3.11	−1.48	0.00	−3.85	−1.69	0.00
Cognitive distress	−4.29	−0.86	0.00	−2.14	−1.17	0.01	−2.82	−1.24	0.00
Intrusiveness	−3.44	−0.76	0.00	−1.86	−0.93	0.01	−2.30	−1.04	0.00
Auditory perceptual difficulties	−2.97	0.18	0.00	−1.12	−0.02	0.00	−1.30	−0.24	0.01
Sleep disturbances	−1.29	−0.14	0.00	−0.85	−0.07	0.00	−0.88	−0.24	0.01
Somatic complaints	−0.56	0.34	0.00	−0.11	0.33	0.02	−0.15	0.24	0.10
**THI**									
Total	−12.93	−1.39	0.00	−19.80	8.38	0.00	−19.78	0.75	0.00
Catastrophic	−2.38	−0.86	0.00	−2.14	−0.55	0.00	−2.33	−0.88	0.00
Emotional	−3.15	−0.46	0.00	−2.36	−0.13	0.00	−3.06	−0.50	0.00
Functional	−3.24	−0.60	0.00	−2.47	−0.28	0.00	−2.88	−0.70	0.00
**TFI**									
Total	−11.26	−2.55	0.01	−14.76	3.67	0.00	−24.25	2.36	0.00
*Intrusiveness*	−7.65	0.64	**n.s.**	−12.05	7.39	0.00	−16.06	3.98	0.00
Control	−14.69	−3.11	0.06	−16.67	3.11	0.00	−30.00	2.76	0.00
Cognitive	−9.75	−0.74	0.04	−11.81	4.50	0.00	−20.10	3.30	0.00
*Sleep*	−4.81	−6.60	**n.s.**	−13.92	−0.32	0.01	−29.29	1.53	0.00
*Auditory*	−5.93	0.54	**n.s.**	−11.23	7.25	0.00	−17.17	4.73	0.00
*Relaxation*	−12.34	−7.82	**n.s.**	−21.46	1.04	0.00	−34.75	0.00	0.00
Quality of life	−16.02	−1.88	0.00	−14.34	2.57	0.00	−22.20	1.07	0.00
Emotional	−17.28	−1.70	0.00	−16.73	4.19	0.00	−25.15	1.90	0.00

*TQ, tinnitus questionnaire (German version); THI, tinnitus handicap inventory; TFI, tinnitus functional index. MCID, minimal clinically important difference. Italicized print indicates subscales with heterogeneous significance levels across the three tinnitus questionnaires.*

Cross-comparing the questionnaires’ responder classifications and associated within-group change scores, responders showed higher levels of change than non-responders did on the [TQ] total and subscale scores, [THI] total and subscale scores, and [TFI] total and “control,” “cognitive,” “quality of life,” and “emotional” subscales. On the remaining TFI subscales, responder change scores differed from non-responder change scores according to the THI and TFI’s but not the TQ’s responder classifications.

### Agreement Between the Measures’ Responders vs. Non-Responders Classifications

#### Kappa

Comparisons of the questionnaires’ responders classifications revealed fair agreement between the TQ | THI (κ = 0.29) and TQ | TFI (κ = 0.27) classifications, and moderate agreement between the THI | TFI (κ = 0.48) classifications.

#### ROC Analyses

*ROC* analyses then estimated the ability of each questionnaire’s [a] baseline-to-post-treatment change and [b] post-treatment scores to distinguish between responders and non-responders as classified by the respectively remaining questionnaires (Table 4).

**TABLE 4:** Change and post-treatment scores distinguishing between responders and non-responders as classified by the respective other two questionnaires.

	**Responders vs. non-responders classification**		
	**TQ [MCID: −12]**	**THI [MCID: −7]**	**TFI [MCID: −13]**
	**AUC (95% CI)**	**AUC (95% CI)**	**AUC (95% CI)**

**Difference scores post-treatment minus baseline**			
^Δ^ TQ		0.69 (0.61 – 0.77)	0.71 (0.61 – 0.81)
^Δ^ THI	0.70 (0.58 – 0.82)		0.83 (0.75 – 0.92)
^Δ^ TFI	0.67 (0.54 – 0.80)	0.85 (0.79 – 0.92)	
**Post-treatment scores**			
TQ		0.55 (0.47 – 0.64)	0.56 (0.46 – 0.65)
THI	0.52 (0.42 – 0.62)		0.55 (0.45 – 0.62)
TFI	0.49 (0.37 – 0.60)	0.52 (0.42 – 0.61)	

*TQ, tinnitus questionnaire (German version); THI, tinnitus handicap inventory; TFI, tinnitus functional index; MCID, minimal clinically important difference; AUC, area under the curve statistic (0.50 < *AUC* < 0.70 low, 0.71 < *AUC* < 0.90 moderate, *AUC* > 0.91 high ability to distinguish between responders and non-responders); CI, confidence interval. ^Δ^, difference score (post-treatment minus baseline score).*

Results indicated that each questionnaire’s total change score “moderately” distinguished between responders or non-responders as classified by the respectively remaining questionnaires. By contrast, post-treatment scores yielded only a “low” ability to do so.

## Discussion

The present study investigated, in the same study, the convergent validity and responsiveness of [a] the German versions of [b] the TQ, THI, and TFI [c] before and after a psychologically anchored, 7-day Intensive Multimodal Therapy Programme. The questionnaires were completed by a large convenience sample of *N* = 210 with chronic tinnitus. Where possible, the present study followed the analysis outline set by Jacquemin et al. (2019) who compared the Dutch versions of the TQ and TFI before and after six sessions of HD-tDCS. Unlike this work, however, our study did not feature a patient-rated clinical global improvement criterion. Consequently, responsiveness analyses were limited to cross-comparisons of the three tinnitus questionnaires.

Across both baseline and post-treatment timepoints, the total scores of the TQ, THI, and TFI showed high convergent validity. In keeping with conclusions drawn by previous studies (Baguley et al., 2000; Jacquemin et al., 2019), all questionnaires thus measure tinnitus-related distress, and their total scores appear comparable across both research and clinical contexts – at least when examining studies from German-speaking populations.

Analogous to results reported by Jacquemin et al. (2019) for the Dutch versions of the TQ and THI, the German versions’ subscale scores showed poor agreement irrespective of similar factor labels. Unlike results from the Belgian study, the [TQ] “cognitive-” and “emotional distress” subscales did not show agreement with the [TFI] “intrusiveness” subscale score thus emphasizing the need to consensually define “intrusiveness” – across both cultural spheres, languages and intervention approaches (Londero and Hall, 2017; Hall et al., 2018). In the present study, the [THI] “catastrophic” subscale showed moderate agreement with the [TQ] “cognitive distress” subscale, the [THI] “emotional” with the [TQ] “emotional distress” and “cognitive distress” subscales and the [THI] “functional” with the [TQ] “emotional distress” and “intrusiveness” subscales suggesting an overlap in measured constructs across these indices. However, a need for homogenization of labels emerges as despite a similarity of measured constructs, applied labels feature wide variability and vice versa (Hall et al., 2016).

Most indices showed significant change with treatment, except for the [TQ] “somatic complaints” and [TFI] “intrusiveness,” “cognitive,” and “auditory” subscales. Unlike results reported following the HD-tDCS intervention (Jacquemin et al., 2019), the more psychologically focused Intensive Multimodal Therapy Programme examined in the present study appeared to be associated with improvements across psychological indices as measured by the TQ, THI and some TFI indices.

Using previously defined MCIDs (Zeman et al., 2011; Meikle et al., 2012; Hall et al., 2018), responder vs. non-responder classifications showed fair agreement for the TQ | THI as well as the TQ | TFI, and moderate agreement for the THI | TFI. Proportionately, the TQ and TFI yielded comparable responder rates (19.65 and 18.64% respectively) whilst the THI responder classification resulted in an overall higher proportion of responders (38.15%).

Investigating change rates across a *scale* × *questionnaire-specific* responders vs. non-responders *classification* matrix revealed that, compared to non-responders, responders showed significantly higher changes across most indices of all three questionnaires. Exceptions comprised the [TFI] “intrusiveness,” “sleep,” “auditory,” and “relaxation” subscales that significantly improved according to the THI’s and TFI’s, but not the TQ’s responders classifications.

Finally, *ROC* analyses revealed that each questionnaire’s change score showed a “moderate-to-high” ability to distinguish between responders and non-responders as classified by the remaining two questionnaires indicating reasonable overlaps in the identification of treatment responders between the three measures. Post-treatment scores yielded only a “low” ability to do so suggesting that [a] all questionnaires adequately measure treatment change and [b] change scores are the index of choice when wishing to quantify treatment change or compare outcome studies.

In conclusion, the present study demonstrated [a] high convergent validity for the total scores of the German versions of the TQ, THI, and TFI and [b] moderate agreement between TQ and THI subscale scores with each discriminating against TFI indices. Each questionnaire is thus suitable as an outcome measure. Baseline-to-post-treatment change scores successfully distinguished between responders and non-responders as per each questionnaire’s responder classification threshold. Comparing the three measures, results of the present study indicated that [a] the TQ and THI showed higher sensitivity to change than the TFI when focusing on statistical significance, [b] the THI and TFI showed higher sensitivity to change than the TQ when comparing responders vs. non-responders as defined by the questionnaires’ MCID scores, [c] the TQ and TFI yielded lower, yet comparable responder rates compared to the THI which classified a higher proportion of patients as responders, and [d] the THI and TFI showed high agreement between responders and non-responders classifications with the former possibly featuring a higher rate of Type I errors. In keeping with Jacquemin et al.’s (2019) conclusion, the TFI appears most suitable as an outcome measure when aiming to identify treatment responders in tinnitus-specific domains. Notwithstanding, the THI or TQ may be preferable when the featured psychological constructs form the focus of interest – perhaps in more psychologically orientated research or intervention contexts.

The present study has important limitations: First, because it did not feature a patient rated criterion of clinical global improvement, the three questionnaires fall short of extended validity or responsiveness investigations. Second, the present two time-point design does not preclude the possibility that measurement error accounted for a proportion of the measured treatment change (Schmidt and Hunter, 1996; de Vet et al., 2006). Future prospective multi-timepoint studies will be helpful in addressing this issue. Third, MCID scores – and thereby responders classifications – are usually established using subjective estimates of clinical global improvement following a particular treatment and are thus likely to show variability across baseline symptom severity, type of intervention, or patient (sub)populations (Olsen et al., 2018; Draak et al., 2019). Fourth, it is noteworthy that the questionnaires’ subscales have not been validated for the assessment of tinnitus-related distress or treatment-related change. Hence, the here-presented subscale analyses ought to be interpreted with caution. Despite these limitations, the present study extends our knowledge of the emerging psychometric literature of measures of tinnitus-related distress by comparing the convergent validity and responsiveness of the German versions of three commonly used questionnaires in the context of a psychologically anchored multimodal treatment programme.

## Data Availability Statement

The datasets presented in this article are not readily available because as per Charité Universitaetsmedizin Berlin’s Ethics Committee, unfortunately, we cannot make the data public without restrictions because we did not obtain patients’ consent to do so at the time. Nevertheless, interested researchers can contact the directorate of the Tinnitus Center Charité Universitätsmedizin Berlin with data access requests (birgit.mazurek@charite.de).

## Ethics Statement

The studies involving human participants were reviewed and approved by Charité Universitätsmedizin Berlin (EA4/137/20). The patients/participants provided written informed consent to participate in this study.

## Author Contributions

BB designed and performed the data analysis, wrote the original draft, addressed the reviewers’ comments, and wrote the final version of the manuscript. BB, PB, and BM supervised data analysis. PB and BM reviewed the manuscript. PB, TK, and BM curated the datasets. BM led the project. All authors contributed to the article and approved the submitted version.

## Conflict of Interest

The authors declare that the research was conducted in the absence of any commercial or financial relationships that could be construed as a potential conflict of interest.
